# Sleep duration in elderly obese patients correlated negatively with intake fatty

**DOI:** 10.1186/1476-511X-11-99

**Published:** 2012-08-07

**Authors:** Aline Alves. Santana, Gustavo Duarte. Pimentel, Monica Romualdo, Lila Missae. Oyama, Ronaldo Vagner Thomatieli Santos, Ricardo Aurino. Pinho, Claudio Teodoro. de Souza, Bruno Rodrigues, Erico Chagas. Caperuto, Fabio Santos. Lira

**Affiliations:** 1 Departamento de Fisiologia, Disciplina de Fisiologia da Nutrição, Universidade Federal de São Paulo-EPM, São Paulo, Brazil; 2 Departamento de Medicina Interna, Universidade Estadual de Campinas, São Paulo, Brazil; 3 Instituto Dante Pazzanese de Cardiologia, São Paulo, Brazil; 4 Departamento de Biociências, Campus Baixada Santista, Universidade Federal de São Paulo, São Paulo, Brazil; 5 Laboratório de Fisiologia e Bioquímica do Exercício, Universidade do Extremo Sul Catarinense, Criciúma, SC, Brazil; 6 Human Moviment Laboratory, São Judas Tadeu University, São Paul, Brazil; 7 Hypertension Unit, Heart Institute (InCor), Medical School of University of São Paulo, São Paulo, Brazil

**Keywords:** Obesity, Sleep, Food intake, Elderly

## Abstract

**Study objectives:**

The purpose of the present study was to evaluate the relationship between sleep duration and dietary habits in elderly obese patients treated at an institute of cardiology.

**Methods:**

The fifty-eight volunteers were elderly patients with obesity (classified as obese according to BMI) of both genders, between 60 and 80 years of age. All participants were subjected to assessments of food intake, anthropometry, level of physical activity, and duration of sleep.

**Results:**

The men had significantly greater weight, height, and waist circumference than women. Sleep durations were correlated with dietary nutrient compositions only in men. We found a negative association between short sleep and protein intake (r = -0.43; p = 0.02), short sleep and monounsaturated fatty acids intake (r = -0.40; p = 0.03), and short sleep and cholesterol dietary intake (r = -0.50; p = 0.01).

**Conclusions:**

We conclude that mainly in men, volunteers that had short sleep duration showed a preference for high energy-density as fatty food, at least in part, may explain the relationship between short sleep duration and the development of metabolic abnormalities.

## Introduction

In human subjects, studies have pointed to the possible involvement of altered sleep hours in the altered energy balance of the body and to alterations in the sleep pattern as a contributory factor to increased obesity [1-3].

Epidemiological and laboratory studies have indicated that self-reported short sleep duration is associated with increased risks for metabolic disruption, including impaired glucose tolerance, impaired insulin resistance, increased ghrelin, decreased leptin, and increased body mass index (BMI) [1,4]. These findings may partially explain the increased mortality associated with short sleep duration, which has been replicated by many studies [5,6]. But the role of diet in these sleep-related metabolic phenomena is currently unknown.

A higher energy intake has been reported in 3-day totally sleep deprived adults [7]. Moreover, early research has suggested a preference for high-fat foods in sleep-deprived humans, [7,8] and recent research in men sleeping 4 h each night for two consecutive nights found that the men had an increased appetite, especially for energy-dense foods with high-carbohydrate contents [4].

Spiegel et al. [9] showed that the appetite for energy-rich nutrients with high carbohydrate content, including sweets, salty snacks, and starchy foods, increased by 33–45%; by contrast, the appetite for fruits, vegetables, and high-protein nutrients was less affected.

Lennernas et al. [10] observed a strong preference for the intake of “fast food” and energy-rich snacks during the nocturnal working hours in night workers. The preference for such foods is a source of great concern since, in addition to presenting a hormone pattern that predisposes subjects to an increased energy intake [9] individuals with sleep loss (common in night workers) tend to meet this need with foods of low nutritional quality [11-15]. This altered food intake can result from inadequate eating facilities during the night shift but, whatever its cause, it increases the risk of obesity [16] dyslipidaemias, [17] and cardiovascular disease [11-15].

In 2002, a large-scale survey was conducted in several countries, including Brazil, with the aim of raising public awareness of the importance of sleep for health, productivity, and safety [18]. In that worldwide investigation, Brazil was classified as the country with the highest prevalence of symptoms associated with sleep disturbances.

Therefore, the purpose of the present study was to evaluate the relationship between sleep duration and dietary habits in elderly obese patients treated at an institute of cardiology.

## Methods

### Sample

This cross-sectional epidemiological study was conducted at the Institute of Cardiology in São Paulo. To be recruited, volunteers had be patient at the Institute of Cardiology, agree to participate in the study; agree to sign the Instrument of Consent; be classified as obese according to BMI (BMI of 30.0 to 39.9 kg/m²), be between 60 and 80 years of age, being in full cognitive and mental condition; not having hearing impairment. Patients on medications such as immunosuppressants, antidepressants, or corticosteroids; and patients with polycystic ovary syndrome, cushing syndrome, hypothyroidism, chronic renal failure syndrome, obstructive sleep apnea, asthma, or depression were excluded.

Fifty-eight volunteers’ elderly (twenty men and thirty-eight women) composed the sample. Twenty-six women volunteers were following dietary, as well as twelve men volunteers were also on dietary treatment. All patients had heart disease, while 45% had hypertension, 38% had diabetes mellitus type II, 69% had dyslipidemias, 3% had stroke, 2% had carotid artery disease, gastritis, arthrosis, bronchitis, heart failure, nephrolithiasis, typical chest pain, and arrhythmia, 10% had impaired fasting glucose, 5% had glucose intolerance, 3% had atrial fibrillation and chronic obstructive pulmonary disease, and 5% had metabolic syndrome and ischemic cardiomyopathy. Full written consent was obtained from all patients and the protocol was approved by both the Institute of Cardiology Committee (4132/2011), and the Federal University of São Paulo Committee (1826/11). Additionally, the protocol is described on the Brazilian Platform for Experimental Research with Human Beings.

### Food intake evaluation

Food intake was determined through Dietary Recall Usual, in which volunteers recall their food intake routinely. The dietary profiles were analyzed using Avanutri® Software.

### Anthropometric evaluation

The anthropometric measures (weight, height, waist circumference, and calculation of body mass index) were assessed from the medical record. A stadiometer installed on the balance was used for the measurement of height. To check weights, a Filizola digital scale with a maximum capacity of 150 kilograms with accuracy of 50 g was used. The waist circumference was measured at the midpoint between the iliac crest and last rib with inelastic tape. BMI was calculated by dividing body weight (in kg) by height (in meters) of the square, according to formula (BMI = body weight/height²). All measurements were performed according to the recommended techniques.

### Physical activity evaluation

To evaluate the degree of physical activity, the questionnaire used was the International Physical Activity Questionnaire (IPAQ). The questions are related to time spent doing physical activity in the previous week. Activities included were: the performing at work, going from one place to another, leisure activities including sport and exercise, or activities at home or in the garden.

### Evaluation of sleep time

Evaluation of sleep time was determined by a questionnaire about nocturnal sleep duration in hours.

### Statistical analysis

Implementation of the Kolmogorov-Smirnov test revealed that the results of experiments were distributed normally. Thus, the variables were expressed by descriptive analysis (mean and standard deviation), and the Pearson correlation was used to examine the associations between sleep duration and food intake (energy, protein, monounsaturated fatty acids, and cholesterol dietary).

The level of statistical significance used was 5% in all tests. For all analyses, we used software STATISTICA version 6.0.

## Results

Fifty-eight volunteers completed all measurements. The characteristics of the participants are presented in Table 1. Male subjects were 65.0 ± 3.7 years of age, and the women were 66.9 ± 4.2 years of age. The mean height and weight for man were 1.68 ± 0.07 m and 95.9 ± 12.5 kg, respectively; in women, they were 1.55 ± 0.06 m and 82.4 ± 9.5, respectively. The mean BMI for men was 33.7 ± 2.8 kg/m^2^, and for the women, it was 33.9 ± 2.6 kg/m^2^. The mean waist circumference of the men was 115.5 ± 8.0 cm, and for the women, it was 108.7 ± 7.1 cm. The mean nocturnal sleep duration for the men was 6.8 ± 1.5 hours, and for women, it was 6.5 ± 1.70 hours. The mean number of kilocalories consumed was 1433.5 ± 472.5 for men and 1200.0 ± 388 for women.

**Table 1:** Parameters anthropometric, physical activity, sleep and dietetic of elderly subjects (n = 58)

**Parameters**	**Mean ± SD**	**Mean ± SD**	**Test t (p value)**
	**Man (n = 20)**	**Female (n = 38)**	
Age (y)	65.0 ± 3.7	66.9 ± 4.2	0.00*
Weight (kg)	95.9 ± 12.5	82.4 ± 9.5	0.00*
Height (m)	1.68 ± 0.07	1.55 ± 0.06	0.00*
BMI (kg/m^2^)	33.7 ± 2.8	33.9 ± 2.6	0.38
Waist circumference (cm)	115.5 ± 8.0	108.7 ± 7.1	0.00*
IPAC	2.3 ± 1.6	2.5 ± 1.0	0.27
Sleep duration (h)	6.8 ± 1.5	6.5 ± 1.70	0.25
Energy caloric (kcal)	1433.5 ± 472.5	1200.0 ± 388.8	0.03*
CHO (g)	183.8 ± 53.5	162.0 ± 62.6	0.08
Protein (g)	77.2 ± 27.5	63.0 ± 30.1	0.03*
Total lipids (g)	43.3 ±28.1	33.1 ± 16.6	0.07
Saturated fatty acids (%)	12.1 ± 8.35	9.38 ± 5.8	0.10
Monounsaturated fatty acids (%)	11.0 ± 8.3	7.4 ± 5.0	0.04*
Polyunsaturated fatty acids (%)	4.7 ± 2.9	4.9 ± 5.1	0.44
Dietary cholesterol (mg)	158. 5 ± 79.0	155.5 ± 93.7	0.45

**Figure 1:**
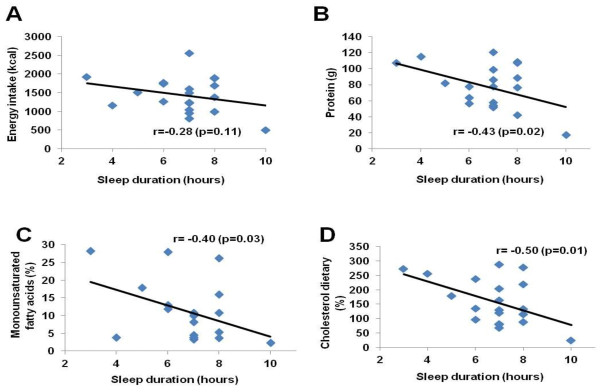
The correlation between dietary and sleep duration in men.

A correlation between sleep duration and nutrient intake was found only in the men and are displayed Figure 1. A negative correlation was found between sleep duration and energy intake for men (r = −0.28; p = 0.11), sleep duration and protein intake (r = −0.43; p = 0.02), sleep duration and monounsaturated fatty acids intake for men (r = −0.40; p = 0.03) and for women (r = 0.05; p = 0.72), and sleep duration and cholesterol dietary intake for men (r = −0.50; p = 0.01) and for women (r = 0.28; p = 0.08). The men had significantly greater weights (14%, p = 0.00001), heights (8%, p = 0.0001), waist circumferences (6%, p = 0.001), and caloric (12%, p = 0.03), protein (22%, p = 0.03), and monounsaturated fatty acids intake (49%, p = 0.04) than women (Table 1).

## Discussion

In the present study, we found links between short sleep duration and increases in energy intake and nutritional consumption of protein and cholesterol. These associations were significant in elderly men but not in elderly women. Here, we examined the impact of diet by usual dietary recall to demonstrate an association between short sleep duration and food consumption and specific nutrients such as dietary cholesterol, protein, monounsaturated fatty acids, and energy intake.

The energy intake in men and women reported implausibly low intakes. A possible explanation may be an underreporting of dietary intake by obese subjects, and also this low energy can be justified, since some voluntary were following dietary.

Numerous studies have documented a high prevalence of underreported energy intakes from-24 hours recalls, food records, and food- frequency questionnaire [19-21].

Underreporting varied according to the dietary assessment method used, but also by the composition of samples from subjects with obesity. Many researchers found that obese people are more prone to underreporting [22]. The few studies on underreporting conducted in developing countries were reviewed and it was found that the proportion of underreporters varied largely from one study and country to another [23].

This hypothesis was confirmed by Scagliusi et al., [24] that underreporting is a prevalent in dietary assessment in a sample of Brazilian women. In another study [25] with subjects as overweight and obesity residing in and around Spanish Town, Jamaica, only about half of the men and the women reported energy intakes within the limits defined as plausible. More than a third of women and nearly a quarter of men reported low intakes. Thus, the underreporting isn´t associated only with dietary assessment method used and composition of samples, but still with country of study.

As reported in other studies, short sleepers have a higher energy intake, suggesting that short sleepers may be more susceptible to weight gain and obesity. The associations found could be explained by appetite hormones, with elevations in serum ghrelin and reductions in serum leptin being partially responsible for the increase in subjective appetite [5,26]. A number of causal pathways linking short sleep duration with obesity have been suggested based on experimental studies of sleep deprivation. One mechanism by which sleep deprivation might predispose subjects to weight gain is by increasing caloric intake. Total sleep deprivation experiments in animals have consistently found that sleep deprivation produces hyperphagia [27]. Partial sleep deprivation experiments in humans suggest a similar effect [28]. Two epidemiologic studies have demonstrated that short sleepers have reduced levels of leptin and elevated levels of ghrelin, supporting the effect of short sleep durations on appetite regulation [5,26].

Additional studies have shown that, more important than an increase in total caloric intake, the proportional intake of specific macronutrients such as carbohydrates or fat [29] as cholesterol, trans-fats and saturated fats could have increased [9].

As expected, analyses of usual dietary recall confirmed the increased preferences for eating energy-dense foods, such as cholesterol-rich foods. Nishiura et al. [30] examined the impact of diet by including questions on dietary patterns, which have been reported to better reflect the association between dietary habit and obesity; [31-33] as expected, self-reporting a preference for fatty food successfully predicted the incidence of obesity, and the preference for fatty food was significantly associated with sleep duration [30].

There is some evidence based on the association between serum cholesterol and saturated fatty acids associated with obesity and metabolic syndrome, which are major risk factors for cardiovascular disease. Study by Waqar et al., [34] demonstrated that the consumption of a normal calorie high-fat diet can lead to insulin resistance and high blood pressure in rabbits, hallmarks of metabolic syndrome, and the consumption of 10% high-fat diet enriched in cholesterol, induced much adverse effects, suggesting that the amount of fat consumed in a diet plays an important role in metabolic disorders and more susceptible to cholesterol-induced metabolic changes : pronounced systemic inflammation, marked elevation of plasma glucose, free fatty acids and insulin, and prominent accumulation of adipose tissue and also demonstrated that the intake of saturated fat with a normal number of calories is detrimental to glucose and lipid metabolism and induces disorders like metabolic syndrome.

It was found in the Seven Countries Study by Ancel Keys [35], the relationship between saturated fatty acids and serum cholesterol that was demonstrated in short- [36] and long-term feeding trials [37,38]; however, a recent meta-analysis of prospective cohort studies showed that the intake of saturated fatty acids is not associated with an increased risk of coronary heart disease, stroke, or the two combined, before [39] or after [40] adjustment or serum total cholesterol. However, in patients who already have cardiovascular diseases, as the patients in this study, the suggestion is that the consumption is within the recommended values.

In present study, 69% of the subjects had exhibited dyslipidemia. Saturated fat and cholesterol intake in men and women surpassed the values currently recommended for this disease, and men may be considered most vulnerable to impairment by cardiovascular disease, since the dietary intake of total lipids, saturated fatty acids, and dietary cholesterol exceeded the dietary intake of women. It is known that dietary recommendations advise the reduction of saturated fat and cholesterol intake for cardiovascular disease risk reduction, with similar recommendations for the treatment of dyslipidemia.

The stronger relationship found between protein intake and dietary cholesterol could be partially justified because both are derived from food of animal origin, which are rich in protein and cholesterol, as well as saturated fatty acids. We suggest that the greater food intake of men with respect to protein was equivalent to their body composition, since men in this study had significantly greater weights, heights, and waist circumferences. Certainly, food consumption is related to food preferences by gender differences.

Although there is a substantial body of evidence that has shown the cardioprotective effects of diets high in MUFA, paradoxical results from experiments in monkeys show that a diet high in MUFA causes atherosclerosis equivalent to that observed in animals fed a diet high in SFA [41]. This effect appears to result from the increased secretion of cholesteryl oleate-enriched lipoproteins. These results, which are counter to the evidence that shows that MUFAs have beneficial effects, need to be further evaluated to determine whether they are relevant to humans. There is also evidence that a fat load provided by olive oil (versus fats high in either SFA or PUFA) increases the plasma levels of chylomicron remnants [42,43] which are atherogenic lipoproteins. However, the preponderance of evidence indicates that dietary MUFAs have favorable effects on CHD risk [44]. However, it is known that the use of such fat provides benefits to patients with cardiac disease and possibly to obese individuals but requires discussions about the high consumption to promote the same benefits as may be associated with a shorter duration of sleep and its complications.

In conclusion, our transversal analysis showed that a preference for fatty food, protein, and monounsaturated fatty acids, at least in part, explains the effects of short sleep duration on the incidence of obesity and cardiovascular diseases, such as dyslipidemia. This study has additional limitations that require discussion. Nevertheless, this result enriches our knowledge of the feeding habits of obese elderly subjects, since this food consumption may favor the onset of co-morbidities associated with short sleep duration and obesity. But without doubt, these findings also motivate us to promote nutrition education and guidance on the importance of quality and duration of sleep and its relationship to health.

## Competing interests

The authors declared no conflict of interest.

## Authors' contributions

AAS, GDP, MR, LMO, RVTS, RAP, CTS, ECC, BR and FSL participated or helped carry out design of the study, sample collected, assess samples, performed the statistical analysis, and writing and discussion of paper. All authors read and approved the final manuscript.
